# Shock, Stress or Signal? Implications of Freshwater Flows for a Top-Level Estuarine Predator

**DOI:** 10.1371/journal.pone.0095680

**Published:** 2014-04-21

**Authors:** Matthew D. Taylor, Dylan E. van der Meulen, Matthew C. Ives, Chris T. Walsh, Ivars V. Reinfelds, Charles A. Gray

**Affiliations:** 1 Port Stephens Fisheries Institute, NSW Department of Primary Industries, Nelson Bay, New South Wales, Australia; 2 School of Biological, Earth and Environmental Sciences, University of New South Wales, Sydney, New South Wales, Australia; 3 Environmental Change Institute, Oxford University Centre for the Environment, University of Oxford, United Kingdom; 4 Batemans Bay Fisheries Centre, NSW Department of Primary Industries, New South Wales, Australia; 5 NSW Office of Water, Wollongong, New South Wales, Australia; 6 WildFish Research, Sydney, New South Wales, Australia; James Cook University, Australia

## Abstract

Physicochemical variability in estuarine systems plays an important role in estuarine processes and in the lifecycles of estuarine organisms. In particular, seasonality of freshwater inflow to estuaries may be important in various aspects of fish lifecycles. This study aimed to further understand these relationships by studying the movements of a top-level estuarine predator in response to physicochemical variability in a large, temperate south-east Australian estuary (Shoalhaven River). Mulloway (*Argyrosomus japonicus*, 47–89 cm total length) were surgically implanted with acoustic transmitters, and their movements and migrations monitored over two years via fixed-position VR2W acoustic receivers configured in a linear array along the length of the estuary. The study period included a high degree of abiotic variability, with multiple pulses (exponentially high flows over a short period of time) in fresh water to the estuary, as well as broader seasonal variation in flow, temperature and conductivity. The relative deviation of fish from their modal location in the estuary was affected primarily by changes in conductivity, and smaller fish (n = 4) tended to deviate much further downstream from their modal position in the estuary than larger fish (n = 8). High-flow events which coincided with warmer temperatures tended to drive mature fish down the estuary and potentially provided a spawning signal to stimulate aggregation of adults near the estuary mouth; however, this relationship requires further investigation. These findings indicate that pulse and press effects of freshwater inflow and associated physicochemical variability play a role in the movements of mulloway, and that seasonality of large freshwater flows may be important in spawning. The possible implications of river regulation and the extraction of freshwater for consumptive uses on estuarine fishes are discussed.

## Introduction

Estuaries represent some of the most variable aquatic ecosystems on earth (e.g. [1]–[3]), but the responses of estuarine species to such variability is often poorly understood [4], [5]. Several sources contribute to the variation often observed in estuarine environments, and these can be anthropogenic or natural. For example, estuary modification can lead to positive or negative changes to estuarine fish assemblages [6]–[8]. Further, natural stressors in the estuarine environment can be induced by rainfall and concomitant changes to river flow, which can alter the physicochemical habitats in an estuary (e.g. through salinity stratification [9]) and increased nutrient inputs into estuaries [10]. The effects of such changes often cascade throughout the trophic chain, either through altered productivity regimes or altered habitat availability (e.g. [11]).

Natural sources of variability in estuarine systems can lead to changes at the scale of hours to years, and are most often associated with variations in freshwater inflow, temperature, tides, wind, and exchange with adjacent coastal waters [12]. In a food-web context, both phytoplankton and nutrients have distinct seasonal cycles [13], and this variability cascades to higher levels in the food chain. In addition, changes to both physicochemical (e.g. [14]) and structural habitats like seagrass (e.g. [15]) can contribute to changes in food webs, foraging habitats, and overall ecosystem structure and function. It is thought that variability in temperature and freshwater inflow are responsible for much of the temporal variability observed in estuary dynamics and species interactions [12].

In the context of fishes, the impacts of freshwater inflow to estuarine systems is often classified into either pulse or press effects [16]. Pulse effects are caused by freshwater pulses, and usually result from large, short-term freshwater inflows which occur as a result of storms and associated run-off, environmental releases of water from storages, unintended over-topping of storages or opening of floodgates. Press effects usually operate over a longer time period, and can arise in response to protracted periods of elevated discharge into estuaries, such as seasonal variation in annual discharge. The impacts of pulse and press events can be either essential or detrimental to fishes life histories. For example, a cyclical or seasonal freshwater inflow may provide a cue to trigger a life history event, such as spawning [17]. Conversely, deterioration in water quality arising from opening of floodgates may lead to osmotic stress, hypoxia and reduced or altered forage resources, which in turn may lead to low survival [18]. The role of freshwater flows as a pervasive stressor, short-term shock, or an important signal for estuarine fish is therefore essential to our understanding of ecosystem processes, regulation, and anthropogenic impacts in estuarine systems.

Acoustic telemetry is an emerging tool which is being increasingly applied in the study of movements of aquatic animals in response to environmental variability (e.g. [19]–[21]). In particular, the development of linear acoustic receiver arrays in estuaries is an extremely useful tool for examining the effects of estuarine inflow on the distribution and movements of fishes (e.g. [22], [23]). This manuscript aims to further explore the links between environmental variability, phenology and fish distribution in highly variable estuarine environments, through acoustic telemetry. Specifically, we examine the effects of freshwater flow, temperature, and conductivity on the horizontal and vertical distribution of a top-level predator (*Argyrosomus japonicus*, hereafter referred to as mulloway) within an estuarine gradient, and explore potential interactions with fish size. This is achieved by evaluating whether abiotic variability correlates with changes in the depth or position of a group of tagged fish along the estuarine gradient, and whether these relationships are consistent amongst different fish sizes.

Mulloway are a common predator in the estuarine and coastal ecosystems of southern Australia, South Africa and China. Juveniles are primarily distributed along the brackish sections of temperate estuaries [24] in deep-hole habitats [25]. Mature mulloway are present in both estuaries and on the open coast, and are thought to undertake both intraestuarine [26] and coastal migrations [27] which may be related to spawning. The extent of interestuarine connectivity, however, is largely unknown. Whilst the species represents a key target for anglers and commercial fishers alike, issues surrounding the sustainability of the fishery have recently arisen both in Australia [28], [29] and elsewhere [30].

## Materials and Methods

### Study Area

The Shoalhaven River (34.90
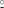
S 150.76
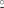
E) is an extensively modified and moderately developed wave-dominated estuary on the New South Wales south coast. The river has a catchment area of 7500 km^2^ [31], which is dominated by agricultural land in the lower catchment and wet and dry sclerophyll woodland (mostly eucalypts) in the upper catchment. The estuary is ≈48 km long, and there is a further 27 km of freshwater between the upper estuary and Tallowa Dam [22]. The mouth of the estuary includes two entrances approximately 5 km apart, including a permanently open entrance at Crookhaven Heads in the south, and an intermittently open entrance at Shoalhaven Heads in the north (which was closed for the duration of the study).

### Ethics Statement

This study was carried out in strict accordance with the recommendations in the *Guide to Acceptable Procedures and Practices for Aquaculture and Fisheries Research, 3^rd^ Edition* [32]. The protocol was approved by the Animal Care and Ethics Committee of the NSW Department of Primary Industries (Permit number 09/01). All surgery was performed under anesthesia, and all efforts were made to minimize suffering. Capture and tagging of fish in the Shoalhaven River during this study was permitted under Section 37 of the *NSW Fisheries Management Act 1994*, through Scientific Research Permit number P01/0059 (issued by NSW Department of Primary Industries).

### Fish Tagging

Thirteen mulloway ranging in size from 47.6–89.0 cm total length (TL) were intracoelomically implanted with Vemco V9 or V13 acoustic transmitters using conventional surgical procedures (e.g. [25], [33]). Briefly, fish were captured from a boat using hook and line, and held in an onboard aerated tank following landing. Prior to surgery fish were bathed in a light anaesthetic (50 mg L^−1^ Aqui-S) until the opercular rate decreased and a loss of vertical orientation was evident. Total length (TL) was measured and fish were placed in an operating cradle for surgery. A 20 mm horizontal incision was made adjacent to ventral midline and a Vemco V9 or V13 tag (Table 1, with some tags containing auxiliary temperature and pressure sensors) was inserted into the coelomic cavity. The incision was closed with two synthetic absorbable sutures (Ethicon Vicryl 3-0) and tied with a double surgeons knot, and an injection of oxytetracycline antibiotic applied at a dose of 75 mg kg^−1^ fish weight. Following surgery, fish were placed in an aerated holding tank to recover, and released at their point-of-capture when they displayed normal opercular and swimming activity.

**Table 1:** Tagging information for mulloway tracked in the Shoalhaven River.

Fish No.	Total Length (cm)	Sex^1^	Transmitter Model	Sensors	Tagging Date	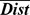 (km)^2^	50%^3^	90%^3^
1	66.0	U	V13-1L	-	25/10/2009	7.1	4.7	10.0
2	68.0	M	V13TP-1L	Temp., pressure	24/11/2009	6.2	14.9	22.3
3	72.0	M	V13-1L	-	24/11/2009	11.5	4.9	11.4
4	80.0	M	V13-1L	-	24/11/2009	3.6	5.1	12.4
5	89.0	U	V13-1L	-	24/11/2009	4.1	11.2	23.7
6	60.0	U	V13-1L	-	25/11/2009	5.5	6.6	29.4
7	82.0	U	V13TP-1L	Temp., pressure	25/11/2009	6.9	1.8	7.2
8	68.0	U	V13TP-1L	Temp., pressure	25/11/2009	3.6	8.1	20.9
9	77.7	U	V13-1L	-	26/11/2009	3.0	4.0	11.9
10	61.7	F	V13-1L	-	26/11/2009	6.6	4.8	8.7
11	55.7	U	V13-1L	-	26/11/2009	6.7	2.7	13.2
12	70.8	U	V13TP-1L	Temp., pressure	26/11/2009	11.8	11.1	23.7
13	47.6	J	V9-2L	-	26/11/2009	28.5	11.0	28.3

1 Sex is male (M), female (F), or juvenile (J). Sex could not be conclusively identified for all samples (U).

2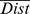 refers to the model distance-to-sea value determined from the kernel density distributions for each fish (see Methods).

3 Linear distance (km) along the estuary encompassed by the 50^th^ and 90^th^ percentile of the kernel density distribution.

### Acoustic Array and Collection of Abiotic Data

**Figure 1:**
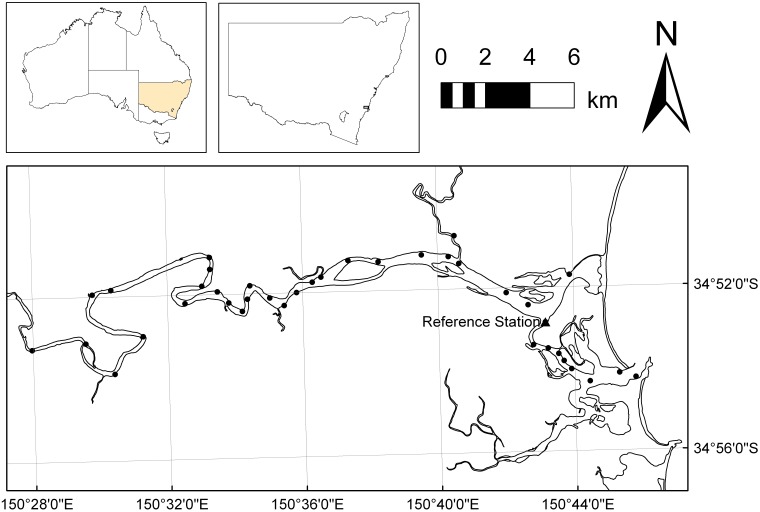
Map of the Shoalhaven River, showing the acoustic array (•) and the temperature and conductivity reference station (▴) from which data was collected for linear modelling.

The Shoalhaven River estuary contains a linear array of 39 Vemco 69 kHz VR2W acoustic receivers [22]. Receiver locations are shown in Figure 1, and covered a 50 km stretch of the river at approximately 1–2 km intervals. Receivers were deployed in an inverted configuration attached to existing navigational markers, as described in Walsh et al. [34], and downloaded quarterly. When a tag transmitted a coded signal in the range of a receiver and was successfully detected, the time and date of detection, identity of the tag, and any telemetered sensor data (e.g. temperature or depth) were logged to the internal memory of the receiver. Receivers had mean detection range of 350 m (range; 280–420 m) in the study estuary [34], and were left in place for the entire study period (November 2010 – January 2012). Previous tracking data within this estuary indicates that there is only a small (0.4%) chance of a fish swimming past a receiver without recording a detection [22].

A series of Odyssey conductivity and temperature loggers (Dataflow Systems Pty. Ltd. Christchurch, New Zealand) were deployed on selected VR2W receivers throughout the estuary, and recorded temperature and conductivity data throughout the entire study period. One of these loggers which was deployed in the area where the greatest density of detections occurred, was selected as a reference station to provide temperature and conductivity data for the analyses. The hourly mean freshwater inflow rates (river flow) into the estuary were measured through the study period at Grassy Gully Creek (NSW Office of Water gauge number 215216, 34.845°S 150.432°E).

### Data Processing and Statistical Analysis

Raw tag detections and associated telemetry data (temperature and depth) were downloaded from VR2W units using the Vemco User Environment (VUE) software v. 1.8.1 (Amirix Systems Inc., Halifax, Nova Scotia Canada), and stored in a Microsoft Access database. Raw data produced in this study are also stored in the Australian Animal Tracking and Monitoring Systems e-Marine Infrastructure Initiative Database (http://aatams.emii.org.au/aatams/). The distance of each receiver station in the linear array to the sea was calculated using ArcMAP v. 10, and matched to each detection in the database to give a distance-to-sea (*Dist*) for each detection. Odyssey logger voltages were processed in Microsoft Excel to provide temperature and conductivity data. Further data processing was performed using MatLab R2012a (Mathworks, Natick, Massachusetts, USA), and tag location and telemetry data were matched to temperature (*Temp*) and conductivity (*Cond*) from the reference station (Fig. 1), and water flow (*Flow*) data. Data processing yielded several composite datasets which were used to explore hypotheses relating to space utilisation (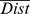, and the 50^th^ and 90^th^ percentile of the kernel density), location in the river (*Dev*), and depth distribution (*Depth*).

Linear kernel density distributions were calculated for each fish from the lateral distance-to-sea (*Dist*) data using the density function in R [35]. Kernel density distributions were used to calculate the modal linear distance-to-sea (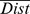) for each fish, and the linear distance encompassed by the 50^th^ and 90^th^ percentile of the kernel density as an estimate of core and total space utilisation vectors respectively. The effect of fish total length (*TL*) on 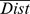 and space utilisation (50^th^ and 90^th^ percentile of the kernel density) was evaluated using simple linear regression.

The relative location in the river (*Dev*) was calculated as the linear deviation of each fish from its model location along the length of the river (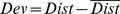). Our main hypothesis related to the relationship between distribution of mulloway along the estuarine gradient and the effects of flow, temperature, conductivity and fish size, and was evaluated using the model:
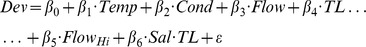
where independent variables reflected those described earlier in the methods, and *Flow_Hi_* was a dummy variable representing high water flow (mean daily flow > highest 5% of flows [36]). *Flow_Hi_* and *Flow* were included to evaluate the pulse and press effects of freshwater inflow to the estuary, respectively. A *Cond·TL* interaction term was included to explore whether relative shifts in distribution in response to seasonal changes in conductivity differed according to fish size. The model fitting process employed generalized least squares (gls) in R v. 2.12.1 (Linear and Nonlinear Mixed-effects Models package [37]) and evaluated the type (auto-regressive [AR], moving average [MA], or both autoregressive and moving average [ARMA]) and order of the error structure which best described any serial correlation in our data (following the approach described in [38]), as well as the best combination of explanatory variables (on the basis of Bayesian Information Criteria). Variables were standardized according to the approach of Kleijen [39]. Significant interaction terms were interpreted using ‘simple slopes’ parameter estimates. To further understand the potential interactions between temperature [40], high freshwater flows (>*Flow_Hi_* [26]), position in the estuary, and spawning, an additional ARMA model was used to evaluate the potential interactive effects of *Flow* and *Temp* on log_10_(*Dist*) of fully-mature fish (>75 cm, n = 3 [28]). The resulting relationships were evaluated in the context of spawning information presented in [28], [41].

To evaluate factors that contributed to a change in depth (*Depth*) of fish implanted with V13TP tags, hourly-averaged depth values were analysed using the model:
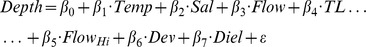
where parameters are as listed above, and *Diel* is a circular representation of diel period calculated from hour-of-day (*h*) (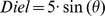, where *ϑ* = 0.26·*h*−1.57). All statistical analyses were performed in R v. 2.12.1 [42].

## Results

### General Observations on Fish Distribution

**Figure 2:**
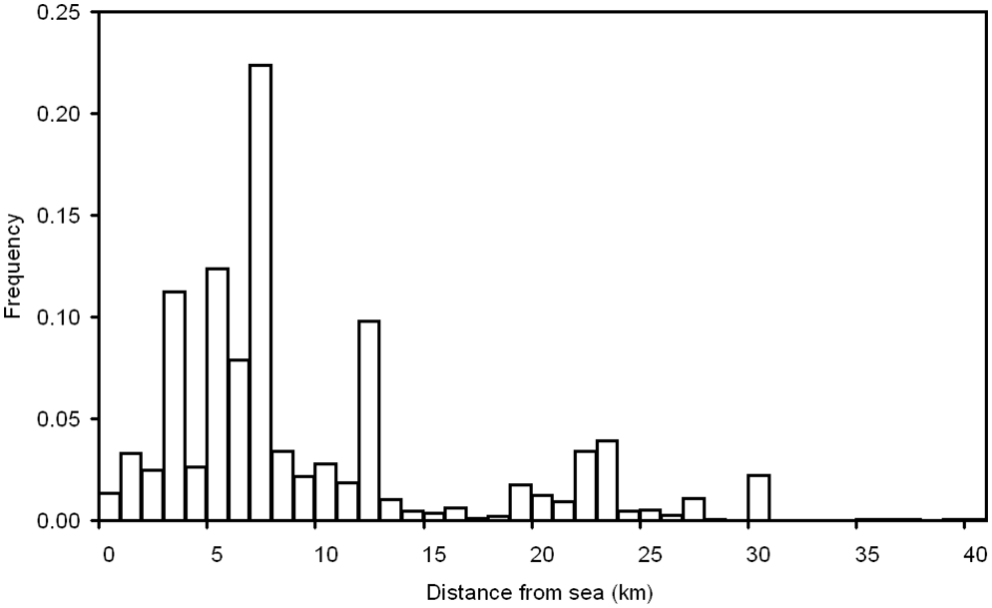
Histogram of measurements for average daily fish distribution along the length of the Shoalhaven River. Data distribution is multimodal with peaks that roughly correspond to 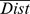 for each fish (Table 1).

The receiver array recorded 257,378 detections over the study period. Fish 5 (Table 1) was only detected in the array for 5 days following tagging, and was excluded from analysis. Fish were detected between the mouth of the river and the VR2W station ≈46 km from the mouth of the river, with a concentration of detections in the lower section of the array (Fig. 2). There was a significant negative relationship between fish total length (TL, mm) and the modal distance-to-sea value (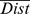) derived from the kernel density estimates (*F*_1,12_ = 5.87, P = 0.03), but not with the total (90%; *F*_1,12_ = 1.13, P = 0.31) or core (50%; *F*_1,12_ = 0.06, P = 0.81) space utilisation distances (Table 1).

### Abiotic Variability

**Figure 3:**
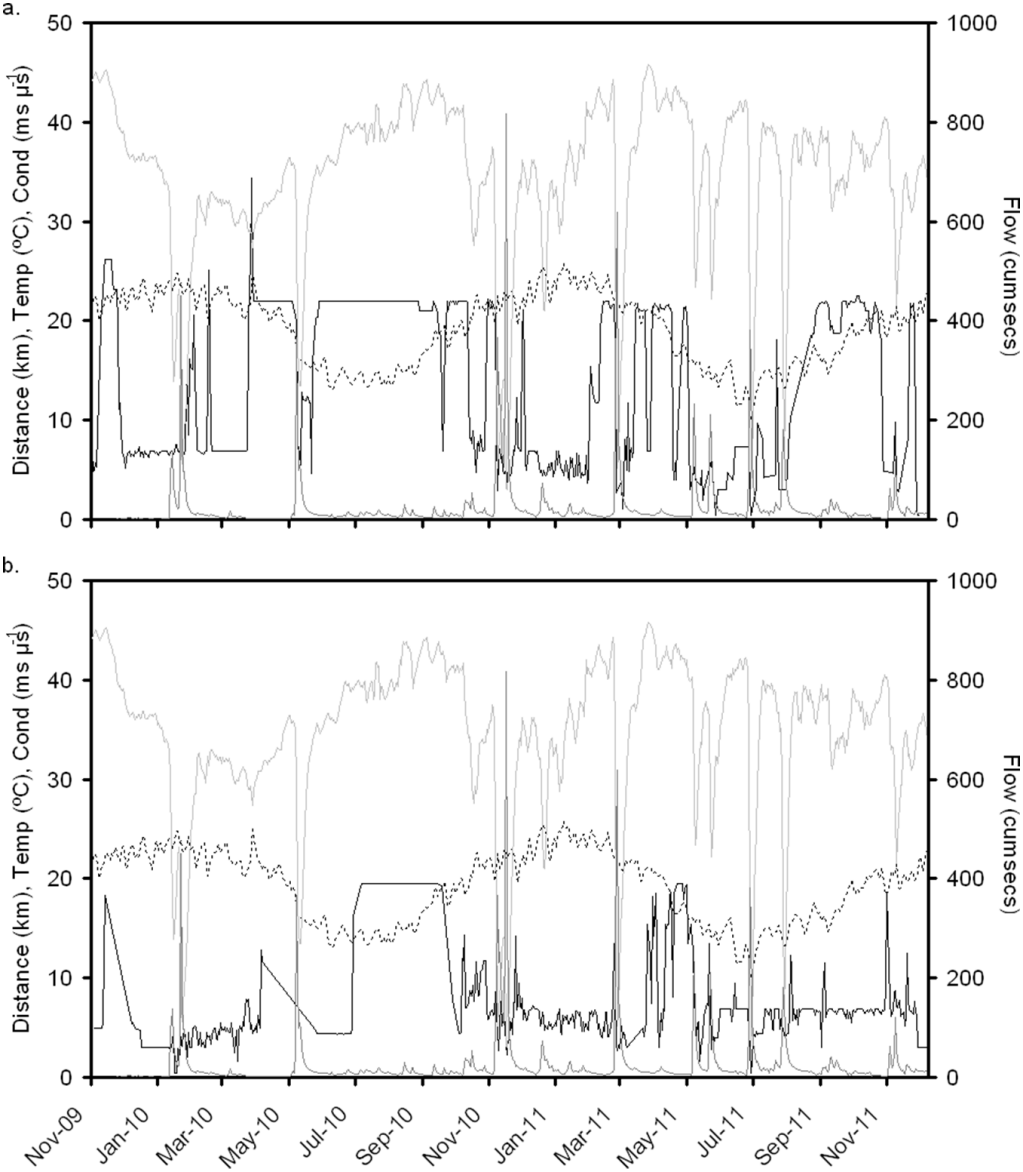
Mean daily position relative to estuary mouth (km), temperature (°C) and conductivity (ms µs^−1^, primary *y*-axis, black line, dashed line and light grey line respectively), and mean daily inflow measured at the Grassy Gully gauging station (secondary *y*-axis, dark grey line) during the study period for Fish 2 (a) and Fish 11 (b).

During the study period, fish were exposed to a high degree of environmental variability, including several moderate to high flow events (Fig. 3a). Conductivity generally decreased sharply in response to high flow events; however, this response was not consistent for all high flow events. Both conductivity (Fig. 3a) and temperature (Fig. 3b) exhibited seasonal fluctuation, and temperature exhibited much less short-term variation during the study period.

### Variation in Fish Distribution

**Figure 4:**
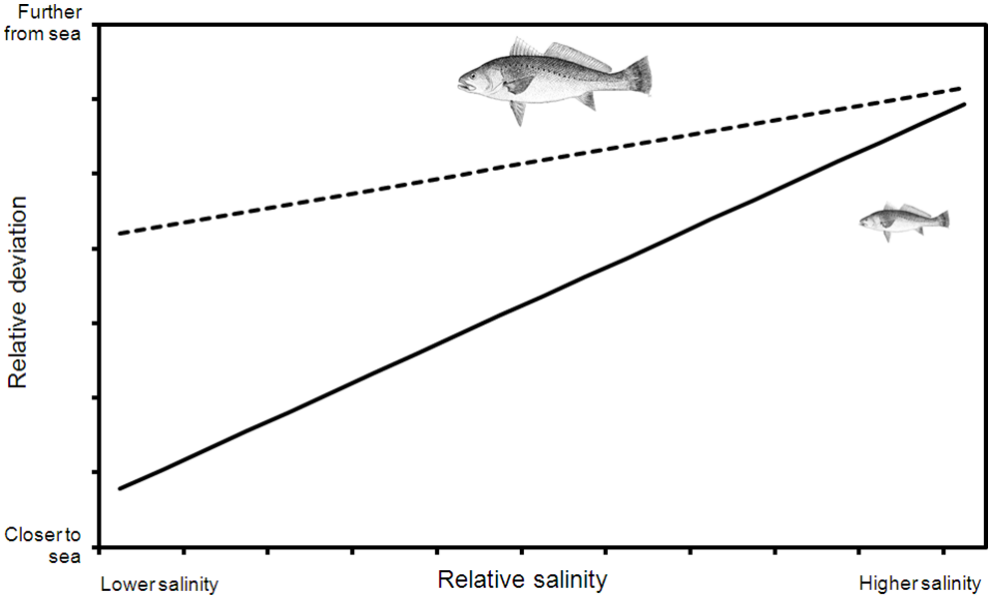
Visual interpretation of significant *Cond·TL* interaction term, showing that smaller mulloway (solid line) display a much more pronounced response to variation in conductivity than larger mulloway (dashed line).

Several models of increasing complexity were evaluated to determine the ARMA structure which best represented serial correlations in the data. On the basis of BIC, characterising the ARMA structure significantly improved the model fit, and a first order autoregressive and second order moving average function provided the most parsimonious model to describe the data (Table 2). The best non-ARMA model indicated that fish size, flow, *Flow_Hi_*, conductivity, and the *Cond·TL* interaction term, were significantly correlated with the relative deviation of fish position within the river (Table 3). After serial correlation was partitioned within the model, the best model indicated that fish length, conductivity, and the *Cond·TL* interaction term, were significantly correlated with relative deviation (Table 3). The significant *Cond·TL* interaction term was interpreted using simple slopes analysis, and indicated that smaller fish (<65 cm, n = 4) displayed a larger deviation from their modal position in the river in response to conductivity (*β* = 0.32, *t* = 5.63, P<<0.01; Fig. 4) than larger fish (>65 cm, n = 8; *β* = 0.19, *t* = 4.32, P<<0.01; Fig. 4).

**Table 2:** Summaries of the full and most parsimonious (best; on the basis of BIC) models trialled for optimisation of ARMA structure, and fitted to the linear deviation of *Argyrosomus japonicus* from its average location along the length of the river (*Dev*).

Model order	*φ*_1_	*φ*_2_	*ϑ*_1_	*ϑ*_2_	Step	Model	BIC
*p = *0; *q = *0	-	-	-	-	Full	*TL* + *Temp* + *Cond* + *Flow* + *Flow_Hi_* + *Cond·TL*	4086.1
*p = *0; *q = *0	-	-	-	-	Best	*TL* + *Cond* + *Flow* + *Flow_Hi_* + *Cond·TL*	4082.6
*p = *1; *q = *0	0.909	-	-	-	Full	*TL* + *Temp* + *Cond* + *Flow* + *Flow_Hi_* + *Cond·TL*	−415.5
*p = *1; *q = *0	0.909	-	-	-	Best	*TL* + *Cond* + *Cond·TL*	−432.8
*p = *2; *q = *0	0.901	0.007	-	-	Full	*TL* + *Temp* + *Cond* + *Flow* + *Flow_Hi_* + *Cond·TL*	−407.7
*p = *2; *q = *0	0.902	0.007	-	-	Best	*TL* + *Cond* + *Cond·TL*	−424.9
*p = *0; *q = *1	-	-	0.762	-	Full	*TL* + *Temp* + *Cond* + *Flow* + *Flow_Hi_* + *Cond·TL*	1784.4
*p = *0; *q = *1	-	-	0.761	-	Best	*TL* + *Cond* + *Flow* + *Flow_Hi_* + *Cond·TL*	1783.6
*p = *1; *q = *1	0.911	-	−0.010	-	Full	*TL* + *Temp* + *Cond* + *Flow* + *Flow_Hi_* + *Cond·TL*	−407.7
*p = *1; *q = *1	0.910	-	−0.009	-	Best	*TL* + *Cond* + *Cond·TL*	−424.9
*p = *2; *q = *1	0.230	0.610	0.712	-	Full	*TL* + *Temp* + *Cond* + *Flow* + *Flow_Hi_* + *Cond·TL*	−407.8
*p = *2; *q = *1	0.235	0.605	0.707	-	Best	*TL* + *Cond* + *Cond·TL*	−424.8
*p = *1; *q = *2	0.941	-	−0.151	−0.047	Full	*TL* + *Temp* + *Cond* + *Flow* + *Flow_Hi_* + *Cond·TL*	−442.5
***p = *** **1; ** ***q = *** **2**	**0.941**	**-**	−**0.152**	−**0.045**	**Best**	***TL*** ** + ** ***Cond*** ** + ** ***Cond·TL***	−**460.2**

The table shows complexity of ARMA structure in terms of AR (*p*) and MA (*q*) parameters included in the model (Order), the auto-regressive (AR, *φ*_n_) and moving-average (MA, *ϑ*_n_) correlation parameters, the terms retained in the model, and the BIC for each model. The best overall model is shown in **bold**.

**Table 3:** Parameter summaries and associated statistics for best non-ARMA model and the best ARMA model presented in Table 2, fitted to the linear deviation of *Argyrosomus japonicus* from its average location along the length of the river (*Dev*).

Model order	Parameter	*β*	S.E.	*t*	P
*p = *0; *q = *0	Intercept	−0.25	0.02	−10.89	<<0.01
	*TL*	0.05	0.02	1.75	0.08
	*Flow*	−0.52	0.03	−15.23	<<0.01
	*Flow_Hi_*	0.09	0.03	3.53	<0.01
	*Cond*	0.06	0.03	1.79	0.07
	*Cond·TL*	−0.32	0.06	−6.76	<<0.01
*p = *1; *q = *2	Intercept	−0.32	0.06	−5.92	<0.01
	*TL*	0.03	0.11	0.26	0.80
	*Cond*	0.23	0.03	7.21	<<0.01
	*Cond·TL*	−0.30	0.07	−4.50	<<0.01

The model evaluating *Dist* of mature fish, indicated a significant *Temp · Flow* interaction term (*β* = −1.64, *t* = −3.08, P<0.01). Interpretation of this interaction term using simple slopes analysis indicated that under conditions of average flow (i.e. mean daily flow <95th percentile of flows), temperature had no effect on *Dist* (*β* = 0.01, *t* = 0.07, P = 0.95); however, conditions of high flow (*Flow_Hi_*) and high temperature were found to have a strong negative effect on *Dist* (*β* = −0.61, *t* = −2.81, P<0.01); indicating that this group of mature fish repositioned themselves much closer to the mouth of the river in response to high temperatures and high flows. High flows and high temperatures tended to coincide with each other during the period December to March (Fig. 3), which coincides with the peak in the number of mulloway in spawning condition sampled in previous studies [28], [41].

### Variation in Fish Depth

Due to the size of the dataset, only non-autoregressive and first order autoregressive functions were fitted to the data. Whilst the best non-ARMA model (BIC = 3597.91) indicated that fish size, diel index, conductivity, and the relative deviation in fish position significantly correlated with relative depth, only the relative deviation in lateral distance to the sea was significantly correlated with relative fish depth (Table 4) after accounting for serial correlation in the data (BIC = −6588.59; *φ* = 0.78).

**Table 4:** Parameter summaries and associated statistics for best non-ARMA model and the best ARMA model presented in Table 4, fitted to the depth of tagged *Argyrosomus japonicus* (Table 1).

Model order	Parameter	*β*	S.E.	*t*	P
*p = *0; *q = *0	Intercept	−0.40	0.01	−60.43	<<0.01
	*TL*	0.15	0.01	24.55	<<0.01
	*Diel*	0.04	0.01	5.39	<<0.01
	*Cond*	0.19	0.02	13.17	<<0.01
	*Dev*	−0.14	0.01	−11.72	<<0.01
*p = *1; *q = *0	Intercept	−0.69	0.01	−81.03	<<0.01
	*Dev*	−0.04	0.01	3.67	<<0.01

## Discussion

Both natural and anthropogenically induced environmental perturbations have a broad range of effects on ecosystems, and contribute much to the ecological variability often observed in estuaries. Agencies dealing in catchment management are increasingly recognising conservation-based objectives in their management plans. Satisfaction of these objectives requires a precise understanding of the consequences of decisions at the catchment level for both species and ecosystems. With respect to river regulation by dams, amelioration strategies such as release of environmental flows and maintenance of environmental variability in estuarine systems is important. At the species level, effects of environmental variability can be both negative and positive, with some species potentially evolving to rely on variability for cues to fulfil certain life history stages. The sustainability issues surrounding mulloway across the species range [28]–[30] necessitate a detailed understanding of the species ecology, so that the species response to both natural and anthropogenic variability can be understood. In this study, acoustic telemetry allowed us to observe a number of press and potential pulse effects of freshwater inflows to a large estuary. The importance and consequence of these effects appears to be partitioned by size, as discussed below.

Long-term monitoring of acoustically tagged fish revealed some likely broad-scale structure within the mulloway population of the Shoalhaven River. Overall, there was a decreasing cline in fish size with increasing distance-to-sea. This is consistent with previous studies which highlight the value of brackish water habitats for mulloway juveniles, which have been deemed important regardless of geographic area [24], [25], [43], [44]. The brackish reaches of temperate estuaries may provide dual benefits for mulloway consistent with the concept of an estuarine nursery (namely abundant food, and lower predation); however, the simple occurrence and use of these areas by juvenile mulloway doesn’t necessarily define it as a “nursery habitat” [45]. It does, however, provide a starting point from which to assess the relative contribution of these habitats to the adult population and thus assess its nursery value [46].

The brackish turbid estuarine transition zone (or estuarine turbidity maxima, see [47]) is often identified as a critical habitat for the development of juvenile estuarine fish, both through retention of larvae [48], and provision of forage resources [49]. Whilst several examples demonstrate the importance of such habitats for early life history stages of Sciaenidae (e.g. [50]–[52]), examples dealing with later juveniles are rarer (e.g. [25], [53]). The broad-scale patterns observed here largely support the findings in these earlier studies on mulloway, albeit over a much longer time-scale. Cowley et al. [53], after Whitfield et al. [54], suggested that a number of biotic and abiotic (e.g. temperature, conductivity, turbidity) factors may impact on the distribution and abundance of mulloway within estuaries. The current study reveals some of the impacts of this variability, with smaller mulloway deviating much further toward the sea during lower conductivities, than their larger counterparts. Whilst high-flow events generally coincided with marked drop in conductivity in this study, the lack of any detectable effect of freshwater pulses (i.e. *Flow_Hi_*) on relative deviation mean that the effect of conductivity may represent more of a press effect of freshwater inflow operating over seasonal cycles.

Recent publications present several hypotheses regarding foraging strategies in mulloway [25], [55]. In South Africa, mulloway tend to ride tidal currents to forage on active teleost prey, such as mugilids and estuarine clupeids [55]. In Australia, smaller mulloway tend to predate on more sessile prey (prawns and shrimp [56]); with movement patterns also reflecting a potential tidal effect of movement [25]. Taken together, however, the cumulative ontogenetic studies on mulloway diet [56]–[59] indicate a clear preference of smaller mulloway for prey taxa (mysid shrimp, penaeid shrimp, and various species of small fish) which are strongly associated with the estuarine transition zone (e.g. [60]–[64]). The overall effects of freshwater inflow in summer tended to push the transition zone further towards the sea (Fig. 3 [65]). Thus, the relationship between conductivity and deviation in the current study is consistent with juvenile mulloway following the seasonal shift in the estuarine transition zone, possibly to facilitate exploitation of prey resources.

Alteration of vertical distribution is a potential strategy that may be employed by fish to deal with environmental variability, whereby fish can seek refuge from low salinities in surface water (during periods of elevated freshwater flow) in bodies of saltier water present at depth. Although there are minimal published studies to support this hypothesis, our modelling indicated that when fish deviate toward the sea, they are more likely to be found in relatively deeper water. There may be multiple contributing factors underlying this observation over and above a refuge from fresher surface water; including the availability of different bathymetric habitats further down the estuary, or altered foraging behaviour in response to the freshwater and concomitant migration toward the sea. It is difficult to further tease out factors contributing to changes in the vertical distribution of tagged fish in this context. Future studies could examine this in more detail by combining telemetry data with bathymetric and habitat mapping, and intensive monitoring of vertical stratification following freshwater flow events.

Whilst some of our results contribute to previous understandings of mulloway movement and space use, the space utilisation results were somewhat divergent from previous studies. This is likely a function of differences in the temporal and spatial extent of monitoring. Taylor et al. [25] found a significant exponential relationship between space use and fish size, with space utilisation ranging between 0.2–0.9 ha (core) and 0.5–1.8 ha (total). Whilst it is difficult to directly compare these estimates with the current study, kernel density estimates from the linear array indicated that space utilisation can span ranges of 2–15 km (core) of 6–29 km (total) of river. Whilst these values are much greater than those previously reported from the manual tracking study (carried out over a lunar cycle), they do reflect the larger estimates reported by Cowley et al. [53] (from <200 days monitoring). The spectrum of estimates derived for this species highlights the importance of investigating movement patterns over a range of temporal and spatial scales in forming a more complete understanding of a species spatial utilisation. It is important to note that our study did not examine movements outside the estuary or between estuaries, both of which will likely influence estimates of space utilisation.

Although there are myriad assumptions in our exploration of a potential spawning signals for mulloway (including a low sample size), our results present several lines of evidence that support previous anecdotal assertions regarding this point (see [26]); namely that estuarine freshwater flows during summer may stimulate aggregations of spawning fish near the mouths of estuaries. A relationship between a freshwater pulse and spawning events could partially explain the observation of a lagged (≈2 y) relationship between large freshwater flow events, and landings of mulloway in the Coorong [66]. Later studies in this system showed strong correlations between freshwater pulses and CPUE [67], with year classes spawned during a high-flow year supporting the catch across the following 8 years. Our study provides some data to underpin a mechanistic explanation of these relationships, which supports the growing body of literature describing the role of freshwater flows as a potential signal for both tropical and temperate species (e.g. [17], [20], [68], [69]). We cannot say that these results are conclusive, and although they do support the model proposed above there are several other potential explanations of these patterns. For example, freshwater flows may provide recruitment cues for coastal larvae and juveniles [69]. Also, terrestrial nutrient inputs which accompany freshwater inflow may contribute to estuarine primary and secondary productivity, which can lead to improved growth and survival of juveniles of exploited species [16]. Furthermore, freshwater pulses may lead to changes in the distribution of teleost prey exploited by larger mulloway [56], [70]. Some investigation of spawning condition of estuarine mulloway directly alongside the monitoring of movements of a wider range of spawning sized fish may help further elucidate the flow-spawning relationship. Clearly, there is much scope for developing a broader understanding of the overall role of physicochemical variability in estuarine processes and fish life cycles in south-eastern Australia.

## Conclusion

This study has revealed several correlations between environmental variability and the location of a range of sizes of mulloway with the Shoalhaven River. It is important to note that our findings are based on a relatively small sample size, but despite this, several patterns were detected which could possibly reflect patterns the wider population. The press effects of freshwater flows (manifesting in reduced salinities) appeared to drive smaller fish closer toward the sea, which may be in response to osmoregulatory stress, or potentially following the shift in the location of the salt wedge within the estuary. The overall effect of freshwater pulses on mulloway was small in our study, but such pulses may provide an important signal for stimulating spawning events for mature individuals and facilitate an aggregation of adult mulloway at the mouths of estuaries. Our understanding of these processes will be improved by examination of similar patterns across other estuaries. River regulation by dams and the capture of flood pulses for consumptive use have the potential to alter estuarine salinity gradients and their location in the estuary, affecting both the intensity of cues experienced by fishes, and their physicochemical habitats. Such regulation may result in a decrease in the frequency of years with high seasonal discharges, which may affect spawning and recruitment success. River regulation and reduced freshwater inflows may also result in a compression of estuarine salinity gradients, reducing the spatial extent of brackish water habitat used by mulloway juveniles. Such impacts may be applicable to a wider suite of species in south-eastern Australia, and other temperate estuaries in the southern hemisphere.
